# Impacts on Coralligenous Outcrop Biodiversity of a Dramatic Coastal Storm

**DOI:** 10.1371/journal.pone.0053742

**Published:** 2013-01-10

**Authors:** Núria Teixidó, Edgar Casas, Emma Cebrián, Cristina Linares, Joaquim Garrabou

**Affiliations:** 1 Ecology Department, Biology Faculty, University of Barcelona, Barcelona, Spain; 2 Institute of Marine Sciences (ICM-CSIC), Barcelona, Spain; 3 Centre for Advanced Studies of Blanes (CEAB-CSIC), Blanes, Spain; National Institute of Water & Atmospheric Research, New Zealand

## Abstract

Extreme events are rare, stochastic perturbations that can cause abrupt and dramatic ecological change within a short period of time relative to the lifespan of organisms. Studies over time provide exceptional opportunities to detect the effects of extreme climatic events and to measure their impacts by quantifying rates of change at population and community levels. In this study, we show how an extreme storm event affected the dynamics of benthic coralligenous outcrops in the NW Mediterranean Sea using data acquired before (2006–2008) and after the impact (2009–2010) at four different sites. Storms of comparable severity have been documented to occur occasionally within periods of 50 years in the Mediterranean Sea. We assessed the effects derived from the storm comparing changes in benthic community composition at sites exposed to and sheltered from this extreme event. The sites analyzed showed different damage from severe to negligible. The most exposed and impacted site experienced a major shift immediately after the storm, represented by changes in the species richness and beta diversity of benthic species. This site also showed higher compositional variability immediately after the storm and over the following year. The loss of cover of benthic species resulted between 22% and 58%. The damage across these species (*e.g.* calcareous algae, sponges, anthozoans, bryozoans, tunicates) was uneven, and those with fragile forms were the most impacted, showing cover losses up to 50 to 100%. Interestingly, small patches survived after the storm and began to grow slightly during the following year. In contrast, sheltered sites showed no significant changes in all the studied parameters, indicating no variations due to the storm. This study provides new insights into the responses to large and rare extreme events of Mediterranean communities with low dynamics and long-lived species, which are among the most threatened by the effects of global change.

## Introduction

Extreme events are rare, stochastic perturbations that can cause abrupt and dramatic ecological change within a short period of time relative to the lifespan of organisms [1], [2], [3]. Extreme events are also considered rapid drivers with the potential to alter the state and trajectory of community structure and dynamics at wide spatial scales [4]–[5], [6], quickly forcing the system away from its equilibrium state and shaping its dynamics far into the future [7], [8], [9]. When ecosystems are forced beyond a threshold, regime shifts occur and the system enters into alternate stable states with a structure and function that are fundamentally different from the previous regime [10], [11]. Thus, understanding the community dynamics affected by extreme events is crucial for ecology and conservation research in a climatically changing world. As a consequence, interest in large phase-shifts and ecosystem resilience related to extreme events has increased considerably during recent decades due to the high level of disturbances that both terrestrial and marine ecosystems are suffering [10], [11], [12].

Studies characterizing marine ecosystem responses to anthropogenic climate change have revealed decreases in ocean productivity, alterations in food web dynamics, changes in physiology, increases in disease incidence, shiftis in species distributions, and reduced abundance of habitat-forming species [13], [14], [15]. In contrast, little is known about how extreme events affect marine communities. Under the conditions of ongoing climate change, observations and global change models predict increases in the frequency and intensity of extreme weather and climatic events, including heat waves, droughts, and intense tropical and mid-latitude storms [2], [16]. Extreme storms, such as hurricanes and severe storms in the tropics and mid-latitude storms in temperate areas abruptly alter ecological processes and structure and severely affect marine littoral communities [17], [18], [19], [6]. In comparison with our understanding regarding the effects of hurricanes and tropical storms affecting coral reefs (*e.g.* [20], [21], [22], [23], [24]), there is little knowledge about how extreme storms affect rocky benthic communities in temperate regions such as the Mediterranean Sea. This lack of knowledge may partially be explained by the rarity and stochastic nature of extreme storms in the Mediterranean Sea, combined with the scarcity of baseline data and long-term studies, making it difficult to study the effects of these events. Nevertheless, analyzing the impacts of these events may provide new insights into processes that shape the structure of benthic communities in this region.

The Mediterranean Sea is considered a hotspot of marine biodiversity, harboring approximately 10% of the world's marine species while covering less than 1% of the world ocean surface [25], [26]. This region has a long history of modification of natural ecosystems by human activities [26]. In the Mediterranean Sea, coralligenous outcrops are of special concern, as they represent one of the most important hotspots for biological diversity (harboring approximately 20% of Mediterranean species), exhibit great structural complexity, and are among the habitats facing major threats [27], [28]. The species that characterize coralligenous seascapes are encrusting calcareous algae, sponges, cnidarians, bryozoans, and tunicates. Some of the engineering species in these environments are long-lived; hence, their low dynamics make coralligenous outcrops exceptionally vulnerable to anthropogenic disturbances, such as destructive fishing practices, pollution, invasive species or mass mortality outbreaks linked to climate change [29], [30], [31], [32], [33]. Moreover, the Mediterranean basin is also considered to represent a climate change hotspot and will undergo one of the largest changes in climate worldwide, with an increase in the frequency of hot wave extremes of 200 to 500% predicted at the end of the twenty-first century [34], [35], [36], [16].

Studies over time provide exceptional opportunities to reveal the effects of extreme climatic events and to measure their impacts by quantifying rates of change at population and community levels. These studies are even more valuable when addressing slow-growing, long-lived species, which do not often undergo marked declines and in which adult mortality is rarely observed [37], [38], [32]. Since 2006, we have annually surveyed coralligenous outcrops in the Medes Islands Marine Reserve in the western Mediterranean, and we were able to detect the impact of a dramatic coastal storm in December 2008 [39], [40], [41] that shifted the community composition and structure of the most common long-lived benthic species in the area. Storms of comparable severity have been documented to occur irregularly within 50 year periods in the Mediterranean Sea [41], [42]. Here, we provide evidence of the immediate impact of this severe coastal storm on the coralligenous outcrops and their responses over the following year. We assessed changes in the dynamics of the benthic community structure using data from before (2006–2008) and after the impact (2009–2010) and by analyzing: i) the community composition, species richness and beta diversity of sessile benthic perennial species with low dynamics, ii) community cover dynamics, and iii) the sensitivity of representative benthic species to the effects of the storm by quantifying cover changes. The final aim of the study is to identify the responses of communities with low dynamics and long-lived species to large and rare extreme events, providing new insights to understand and predict how present and future impacts affect these communities.

### Extreme Storm Event on December 26^th^ 2008

The December 26^th^ 2008 storm was an extreme event considered to be one of the strongest impacting the Catalan coast in the last 50 years [43], [41],[42]. Storms of an equivalent intensity were reported for the same area in the early (31/01/1911) (Meteorological Service of Catalonia, http://www20.gencat.cat/docs/meteocat/Continguts/Noticies/2011/Gener/pdf/31degenerde1911.pdf), [44] and mid-twentieth century (22/02/1948) (La Vanguardia newspaper archives, http://hemeroteca.lavanguardia.com/preview/1948/02/22/pagina-4/34354259/pdf.html); but there are no instrumental wave records of these storms. On December 25^th^ 2008, a strong high pressure system developed over northern Europe (1047 hPa) blocking the western atmospheric circulation and forcing northern cold air and a deep cyclone to flow towards the NW Mediterranean Basin [41], [39]. This convergence caused maritime eastern winds and stormy seas to reach the Catalan coast. The storm reached category 5 [45] as it moved from the Gulf of Genoa to the Catalan coast, where it hit the shore on December 26^th^, with wind gusts up to 20 m s^-1^, wave heights of 8 m with peaks of 14.4 m, and wave periods of 14 s [39], [41]. The damage caused by the intense waves was accentuated by the dislodged material that they carried, scouring sand and the displacement of large rocks [41], [N. Teixidó *pers. observ]*. Shallow sublittoral communities (5–10 m) in the Natural Park of Montgri, Medes Islands and Baix Ter and adjacent areas showed high abrasion, with density reductions of 50–80% of sea urchin populations and loss of algal cover up to 90% within a depth range of 5–10 m [46],[47]. Although these shallow habitats were the most impacted, damage was also registered in deeper habitats (20 m depth), with 80% of mortality of the brown alga *Cystoseira zosteroides* [40]. The most damaged communities were dwelling on surfaces facing the East. An exploratory dive immediately after the storm at a depth of approximately 16–20 m (one week afterward, 03/01/2009) revealed detached living colonies and fragments of gorgonians (*Paramuricea clavata* and *Eunicella singularis*) and massive sponges (*Ircinia oros*) on the sea floor, torn skeleton bases with living tissue of *P. clavata,* rhizomes of *P. oceanica*, and displacement of large rocks with compressed sessile organisms (N. Teixidó *pers. observ.*). Additionally, the storm caused a significant decline of the sea bass population (*Dicentrarchus labrax*), the burial of 20% of *Posidonia oceanica* meadows and affected the deep-sea environment (300–1500 m) through increases of current speed, sediment transport, and the grain size of particles [43],[41].

## Materials and Methods

### Study Area

We assessed the impact of this dramatic storm on coralligenous outcrops in the Natural Park of Montgri, Medes Islands and Baix Ter of the NW Mediterranean Sea (42° 3′N 3° 13′E, NE Spain). This area harbors well-developed coralligenous outcrops with a depth distribution of 15–70 m [48]. Due to their beauty and aesthetic value, these outcrops are among the most attractive areas for recreational scuba diving and are subjected to diving impacts [49], [30].

### Field Activity and Data Collecting

**Figure 1:**
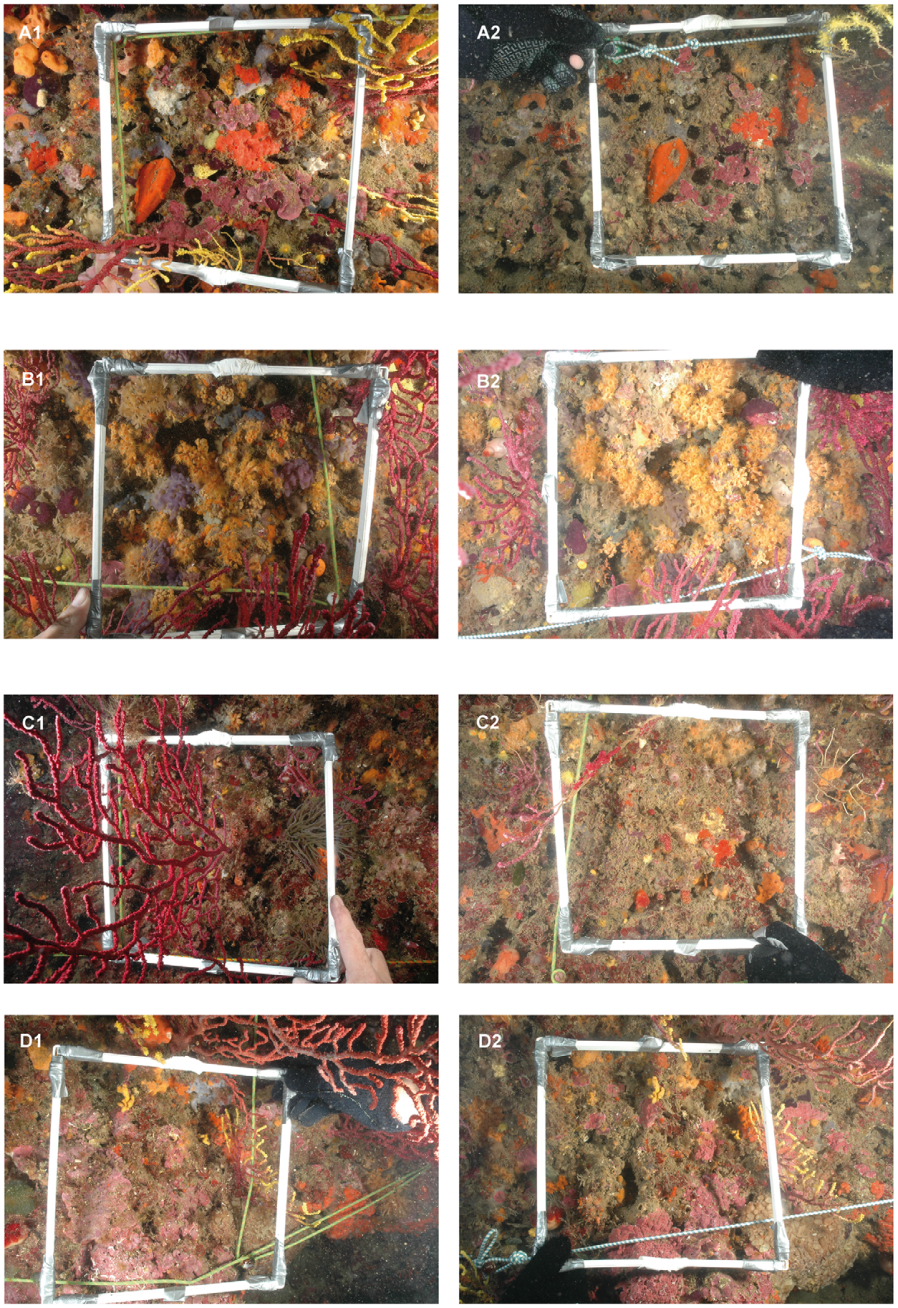
Photographs showing the impact of the coastal storm on coralligenous outcrops. These photographs show the same quadrat (25*25 cm) before (2008) and after the storm event (2009). A: Carall Bernat, dates A1∶23/06/2008, A2∶10/02/2009; B: Tascó Petit dates B1∶24/06/2008, B2 16/02/2009; C: Medallot, dates C1∶22/06/2008, C2∶09/02/09, D: Punta Salines dates: D1∶25/06/2008, D2∶09/02/2009.

We quantified the immediate impact of the storm on the benthic community and the following year using before-and-after data (Fig. 1). Sampling site locations had different exposure orientation, where the most exposed face the East and the most sheltered the North-West: Carall Bernat faces the NE, Medallot the SW, Tascó Petit the NW, and Punta Salines the N. The sites are separated by few hundreds of meters to 3 kilometers. Carall Bernat was the site most exposed, whereas Tascó Petit and Punta Salines were the most sheltered; thus used as controls. We present data from surveys that were performed annually before the storm event (July –August 2006, 2007, and 2008), shortly after the storm (February 2009) and one year later (August 2010). Data available from Punta Salines cover only 2008 and February 2009. However, the Punta Salines data set has a meaningful ecologic value because it covers the most relevant time span of the analyzed temporal variation (before and immediately after); thus, we considered as a valid control site. This severe storm was a natural experiment affecting sublittoral communities with differences in exposure among sites and offered the possibility to reveal the effects produced after this severe meteorological event.

We used data from 4 permanent plots (4 m long * 0.8 m wide, total area  = 3.2 m^2^) located haphazardly at a depth of ∼ 20 m at four different sites. The corners of each plot were marked with PVC screws fixed in holes in the rocky substratum with two-component putty [37]. During each survey, elastic bands were placed around the corners to facilitate the recognition of plot borders. Then, each plot was monitored photographically using quadrats of 25*25 cm to facilitate species identification [50]. The photographs were taken with a Nikon D70S digital SLR camera fitted with a Nikkor 20 mm DX lens (3000 * 2000 pixel resolution) and housed in Subal D70S housing. Lighting was achieved using two electronic strobes fitted with diffusers. Approximately 64 quadrats covered the entire surface of the permanent plot. In each permanent plot, we analyzed 3 replicates of 8 photographic quadrats (5000 cm^2^) as a minimal sampling area as the optimal sampling effort [50]. These 3 replicates per site (n = 51 in total: 3 sites* 3 replicates* 5 years  = 45; 1 site * 3 replicates* 2 years = 6) allowed replication and further statistical comparisons. A total of 404 photographs were analyzed.

All necessary permits for the described field studies were obtained from the authority responsible for this Protected Area. The locations are not privately-owned. This study did not involve endangered or protected species. Moreover, we did not perform any disturbance to species during our fieldwork. Our data were based on the analysis of images, a non-destructive technique to study marine benthic communities.

### Benthic Communities

Natural variability of coralligenous outcrops shows little changes over time [51], [32], [52]. Changes in the benthic coralligenous outcrops due to the severe storm were evaluated in three ways. i) Changes in community composition, species richness, and beta diversity were measured based on the presence-absence of perennial sessile macro-species. Overall, these perennial species are characterized by slow growth and low dynamics (hereafter referred to as SG, see Table S1 for the species list) [53], [54], [55], [32]. They mainly consisted of macroalgae and encrusting red algae, sponges, anthozoans, bryozoans and tunicates. A total of 64 SG species were identified at the lowest taxonomic level from photographs. Additional dives were performed for species identification (see [50] for further details). This approach, based on presence-absence analysis is an optimal method for coralligenous biodiversity assessment and monitoring, providing good estimates of the composition and structure of these communities [50]. ii) The percent cover of functional groups of sessile organisms and substrates was measured. These groups were classified as slow growing species (hereafter SG, i.e., the 64 species mentioned above with low dynamics); fast growing species (hereafter FG, including small, filamentous and seasonal hydrozoans and bryozoans with high dynamics); turf of algae (hereafter TA, corresponding to a multispecific assemblage of small and filamentous algae); detritic matrix (hereafter DM, consisting of conglomerates of detritus and microalgae); and bare substrate (hereafter BS). iii) The percent cover of the representative slow growing species was determined by the similarity percentage procedure (SIMPER analysis) (see below). Then, the 37 representative species were grouped into 6 different morphological forms: Boring (BOR), Cup (CUP), Encrusting (ENC), Encrusting algae (ENA), Massive (MAS), and Tree (TREE). Furthermore, we measured the sensitivity of these 37 representative species by comparing the change in the percentage of cover before and after the storm (see Table S2 for cover values). The sensitivity values ranged from −100% (total disappearance of cover after the storm) to 0% (no cover change) to positive values (increased cover). To perform the cover analyses, each photograph was projected onto a grid of 25 squares (5 cm×5 cm), and abundances were quantified by counting the number of squares filled in the grid by either each functional group or representative species and expressing the final values as percentages [56], [57]. For the red gorgonian *Paramuricea clavata,* which exhibits an arborescent form, cover was calculated as the area occupied by its base. Percent cover of functional (ii) and morphological (iii) groups over 5 years were calculated for sites where all temporal range was available.

### Statistical Analysis

Changes in community composition were investigated using non-metric multidimensional scaling (MDS) on the basis of the Bray-Curtis dissimilarities of the presence-absence of 64 perennial macro-species as well as the presence-absence of the functional groups described above (FG, TA, DM, BS). The null hypothesis of no structure in the data was tested using the similarity profile test (SIMPROF) (with 9999 permutations and a 0.1% significance level [58] on the Bray-Curtis matrix). This technique is a permutation-based ranking procedure aimed at testing genuine clusters in samples with no *a priori* assumptions about group membership. Differences in beta-diversity (% of unshared perennial macro-species) among sites and before and after the impact were analyzed using the PERMDISP routine. This is a routine for comparing the degree of dispersion of different groups of samples based on a distance matrix. We tested for similarity in the beta-diversity among groups on a Jaccard distance matrix [59]. The representative taxa for each site before the storm were determined using the similarity percentage procedure (SIMPER) [60]. Then, we measured the sensitivity of these taxa by comparing the percentage of cover change before and after the storm.

Non-parametric analysis of variance PERMANOVA [61] was used to examine the changes generated by the storm. The sampling design included 2 factors: *Site*, which was random with 4 (changes in community composition) or 3 (cover of functional and morphologic groups) levels; and *Before/After*, which was fixed with 2 levels. Differences between samples were quantified using i) Bray-Curtis dissimilarities for the multivariate perennial macro-species data matrix and ii) Euclidean distances for univariate analyses. Analyses were performed with 9999 unrestricted random permutations of the raw data. Pair-wise comparisons for all combinations of *Site x Before/After* were also carried out using t-tests and 9999 permutations of the raw data. Chi-squared tests were carried out to test for differences in the frequency of sensitivity among the sites and taxonomic as well as morphological form groups. The analyses were computed using the program Primer v6 with the PERMANOVA+add-on package [62] and Statistica (version 8.0 StatSoft).

## Results

**Figure 2:**
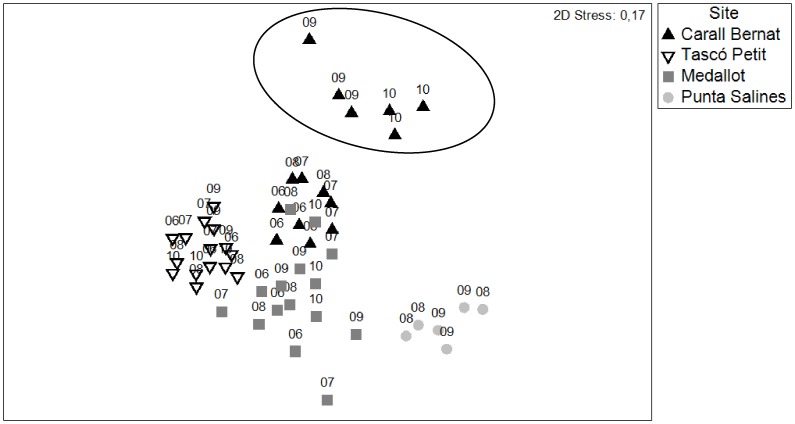
Shift in the structure of the perennial benthic species in coralligenous outcrops in the Medes Islands in response to the 2008 dramatic storm episode. Non-metric multidimensional scaling (NMDS) based on the Bray-Curtis resemblance measure for species presence/absence data from 2006 to 2010. The circle indicates a SIMPROF group containing the immediate post-storm (2009) and following year (2010) data for Carall Bernat. Each symbol represents 8 analyzed photographs.

**Figure 3:**
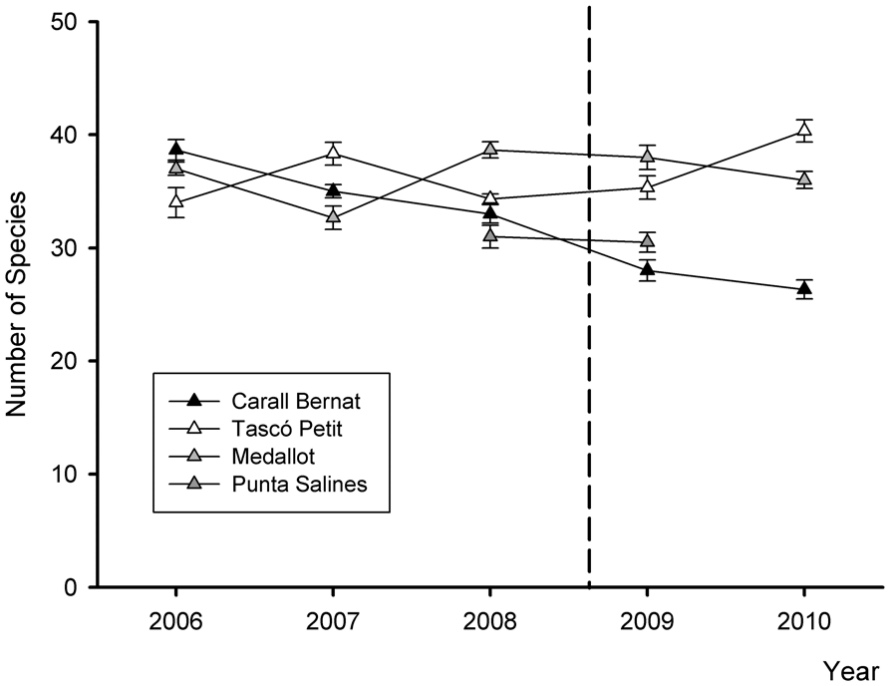
Mean number of sessile species (± SE) over time in the Medes Islands. The dotted line represents the impact of the unusual storm in December 2008.

**Figure 4:**
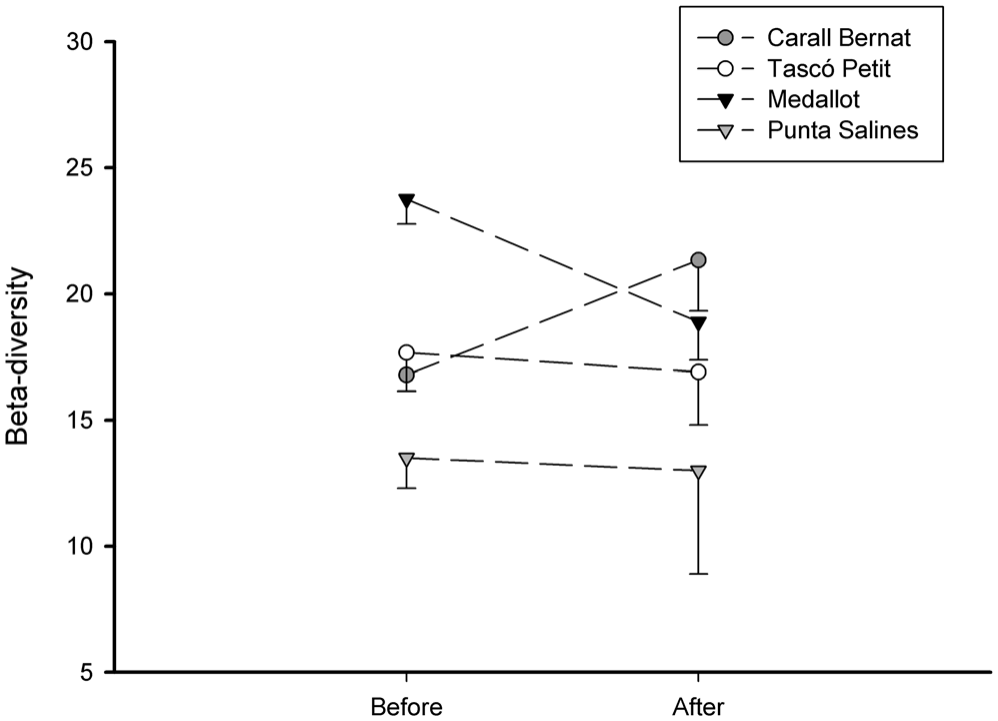
Mean (±1 SE) beta-diversity (as the percentage of unshared species) before and after the storm for each site. The results of PERMDISP analyses are shown.

The community composition of sessile macro-species showed a major shift after the immediate impact of the storm (Fig. 2). The most exposed site Carall Bernat was the most impacted and underwent a change in benthic structure, resulting in a distinct cluster (SIMPROF test p<0.01, see Figure S1 for all the SIMPROF groups) containing the immediate post-storm (2009) data and those of the following year (2010). This post-storm group showed a higher dissimilarity and larger multivariate dispersion than the pre-storm data (2006–2008) and those from the other three sampling sites; no significant changes were observed (Fig. 2). There was a significant interaction between sites and before-after the storm impact (F_3,43_ = 2.96, p<0.0001) (see Table S3 for 2-way PERMANOVA and pair-wise tests). Considering the pair-wise comparisons, only Carall Bernat showed a significant difference before (85% similarity) and after the storm (74% similarity) (t = 2.98, p<0.0001, Table S3). This shifting pattern was corroborated by a significant decrease in the mean species number (F_3,43_ = 8.91, p<0.001), from the mean values of 35.5±0.57 before to 27±0.62 sessile species after the storm, representing a decline of 24% (pair-wise comparisons t  = 5.99, p<0.0001) (Fig. 3) (see Table S4 for 2-way PERMANOVA and pair-wise tests). The clear shift in the community composition was also evident based on the beta-diversity analysis (F_7,43_ = 5.8, p<0.001) (Fig. 4). Carall Bernat showed a significant increase of beta-diversity after the storm (18.2% ±0.7 before vs. 22.33% ±1.4 after) (t  = 1.78, p<0.01), indicating higher variation in the benthic composition, whereas Medallot exhibited a decrease, which was not significant (24.4% ±1.2 before vs 18.7% ±1.4 after) (t = 2.97, p>0.05). Regarding the non- impacted sites, no changes were observed in the community structure (Fig. 2, Table S3), mean species number (before: Tascó Petit 34.3±0.43, Punta Salines 31.1±1.1; after: Tascó Petit 35.3±1.0, Punta Salines 30.5±0.8) (Fig. 3, Table S4) and beta diversity (Fig. 4) (t = 0.9 p>0.05 for Tascó Petit, t = 0.9, p>0.05 for Punta Salines).

**Figure 5:**
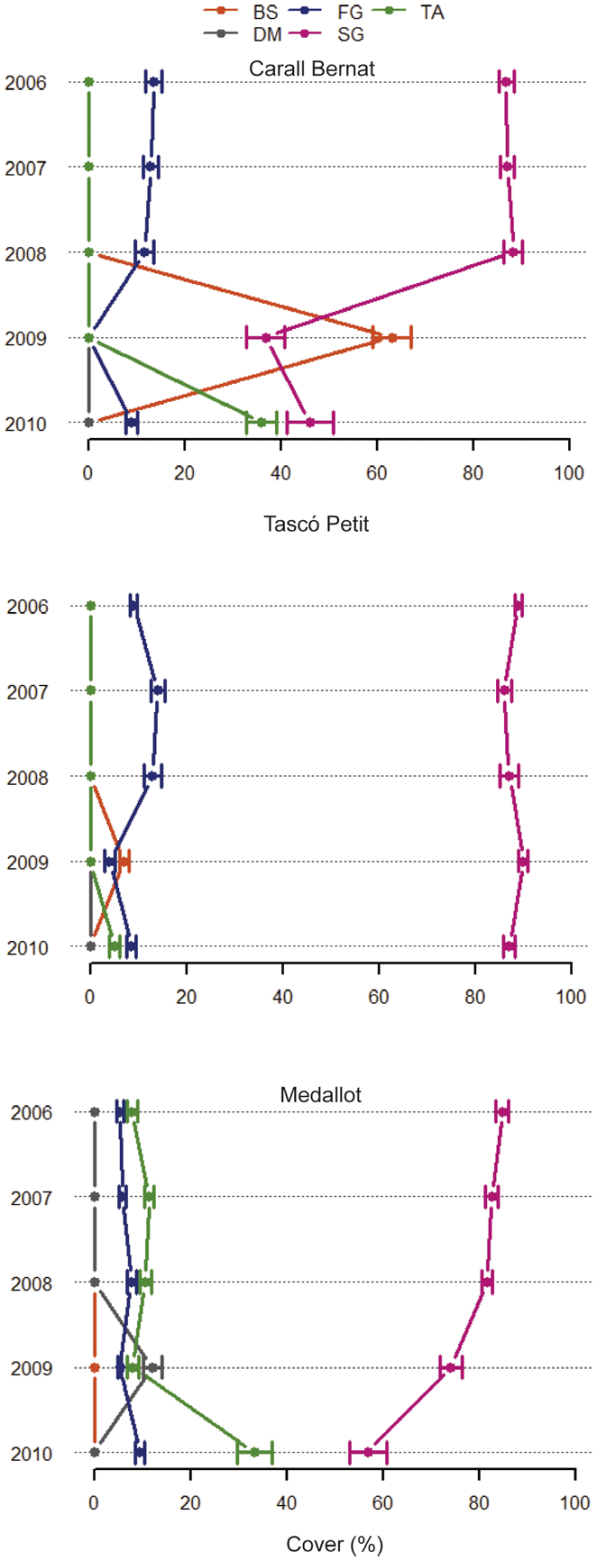
Response to the dramatic 2008 storm episode regarding the cover area of the principal groups of sessile organisms and bare substrate. The principal categories are Bare substrate (BS), Detritic Matrix (DM), Turf algae (TA), Fast growing species (FG, i.e., small animal species mainly bryozoans and hydrozoans), and Slow growing species (SG, i.e., perennial algae and animal species).

**Figure 6:**
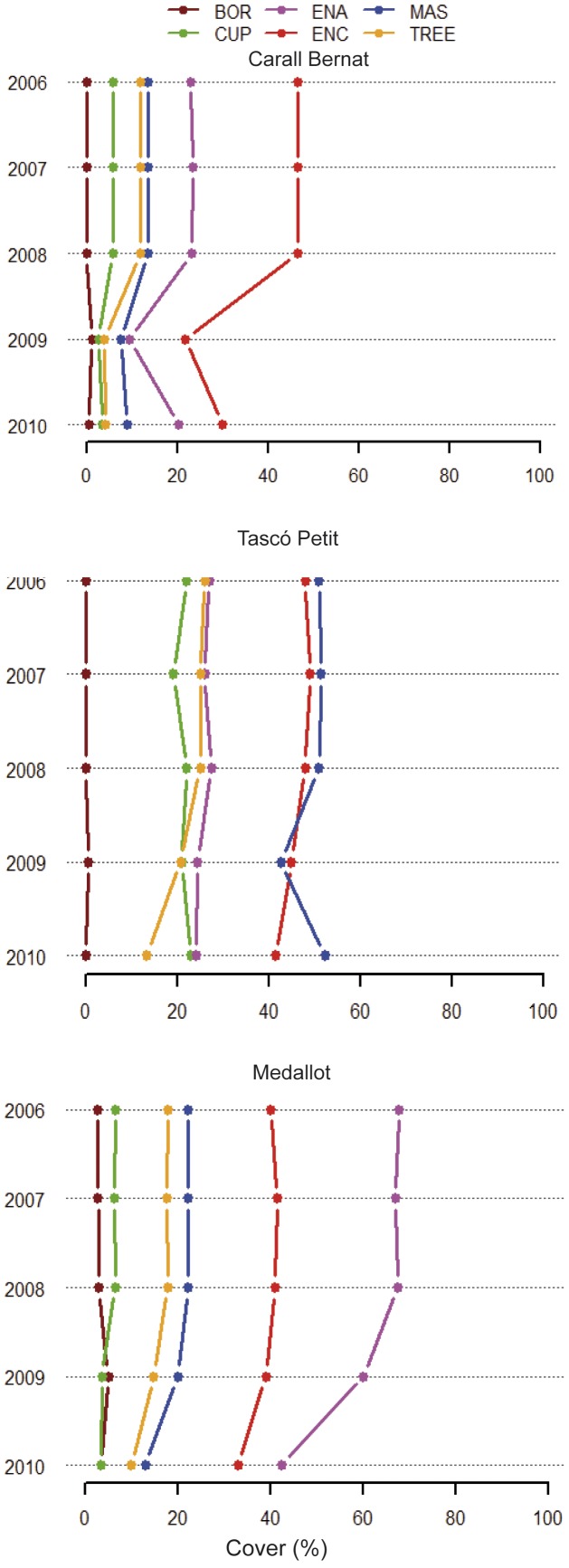
Response of the cover area of the representative macrobenthic taxa to the dramatic 2008 storm episode. These taxa account for 90% of the average similarity within each site (SIMPER analysis). The taxa are represented as 5 morphological forms: Boring (BOR), Cup (CUP), Encrusting algae (ENA), Encrusting (ENC), Massive (MAS), and Tree (TREE).

Before the storm event (2006–2008), the coralligenous outcrops were characterized by a high cover of perennial and slow growing species (SG) (mean values of 87% ±0.9 Carall Bernat, 88% ±0.8 Tascó Petit, and 84% ±0.7 Medallot) (Figs. 5 and 6), such as encrusting and fragile calcareous algae, encrusting sponges, tree bryozoans and gorgonians, massive sponges and tunicates, and an overall high structural complexity. These patterns were constant over the three years and reflected the low natural variability of coralligenous outcrops (Fig. 5). There was a significant change in the percentage of cover of the principal functional groups among the three sites and before-after the storm (F_2,39_ = 10.7, p<0.0001) (Fig. 5), and these differences were significant for the pair-wise comparisons of the interaction term at Carall Bernat and Medallot (p<0.001, see Table S5). The cover of SG at Carall Bernat was by far the most severely damaged, showing a decrease to 37% ±3.9 of the total area immediately after the impact (2009), but increased to 46% ±4.7 in the following year (2010) (Fig. 5). The distribution of damage also depended on small-scale position effects at Carall Bernat, where 16% and 25% of the area analyzed in 2009 showed values as low as 10% and 50% of SG cover, respectively. The scouring effect of the storm was evident in the peak of bare substrate (BS) (mean value 63% ±4) observed immediately after the storm (2009) at Carall Bernat, which was replaced by turf algae (TA) in 2010 (mean value 44% ±3). Interestingly, surveys at Medallot showed a moderate decrease of SG, with a reduction from 84% ±0.7 (before) to 74% ±2.3 (2009), followed by a further decrease down to 56% ±3.8 (2010), suggesting a delayed loss of SG cover (Fig. 5). Furthermore, there was an increase of detritic matrix (DM) in 2009 (mean value 12% ±0.2) and a 3-fold increment from the pre-storm cover value of TA (mean value 33.3% ±3.6 in 2010). In contrast, Tascó Petit was almost not affected after the storm, exhibiting a discrete peak of 5% BA and a reduction of 66% in fast growing species (FG) (12% ±1.8 before vs. 4% ±1 after) (Fig. 5). No significant change in cover percentage of the principal functional groups was observed before and after the storm (pair-wise comparisons t = 2.63; p>0.05, see Table S5).

**Figure 7:**
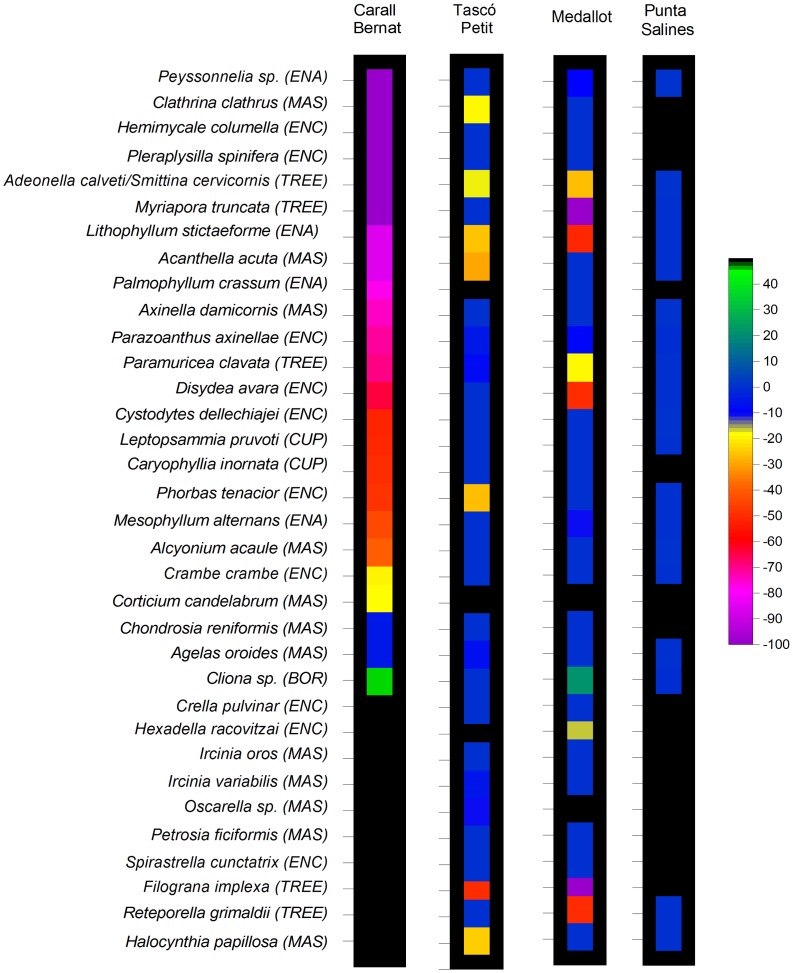
Sensitivity (as a percentage of cover change) of the representative macrobenthic taxa to the physical disturbance generated by the storm. Cover change (from highest, −100%, to lowest, 0%) at Carall Bernat defines the order of the taxa at the other sites. Representative macrobenthic taxa were chosen from SIMPER analysis. Black areas indicate that taxa were not representative for the specific site.

The damage to the cover of different growth forms differed significantly among localities and before-after the storm (Fig. 6) (F_2,9_ = 3.7, p<0.01) The cover loss of massive (MAS) and encrusting (ENC) sponges and tunicates, scleractinian corals (CUP), encrusting algae (ENA), and arborescent gorgonians and bryozoans (TREE) ranged from 45% to 66% in relation to the pre-storm cover at Carall Bernat (Fig. 6). The damage to ENC and ENA was particularly striking due to the high pre-storm cover (approximately 46.5% and 23% before and 21.8% and 9.5% after the storm, respectively), as well as for the TREE category, which despite its low cover before the storm (approximately 12%) declined considerably to 4% (Fig. 6). Medallot and Tascó Petit did not show significant differences before and after the storm (pair-wise comparisons t  = 2.13 and t = 2.45, p>0.05, see Table S6). The sensitivity of the representative species most affected by the storm was significantly different across sites, with 95% of species being affected at Carall Bernat (n = 24), 38% at Tascó Petit (n = 31), 34% at Medallot (n = 32), and less than 1% at Punta Salines (n = 19) (*X^2^* = 54.2, df = 3, p<0.0001) (Fig. 7). The alga *Peyssonnelia sp*., the encrusting and delicate sponges *Hemimycale columella* and *Pleraplysilla spinifera*, the massive-ropy fragile sponge *Clathrina clathrus* and the bryozoans *Adeonella calveti* and *Myriapora truncata* were reduced by up to 100% at Carall Bernat (Fig. 7, Table S2). In addition, among the species that exhibited high coverage before the storm (each species showing a cover value of approximately 10%), *Lithophyllum stictaeforme* (ENC) was reduced to 85%, *Parazoanthus axinellae* (ENC) to 72%, *Paramuricea clavata* (TREE) to 70%, *Disydea avara* (ENC) to 64%, *Phorbas tenacior* (ENC) to 49%, and *Crambe crambe* (ENC) to 20%. Similar patterns of damage were not found at the other sites. For example, at Medallot, the reduction was 100% only for *F. implexa* and for *M. truncata* (TREE*),* and other species showed values lower than 50% such as *D. avara* (ENC), *Reteporella spp.* (TREE), and *P. clavata* (TREE). Only 3 species out of 31 showed high to moderate values of cover loss at Tascó Petit: 100% for *Halocynthia papillosa* (MAS), 67% for *C. clathrus* (MAS), and 50% for *Filograna implexa* (TREE). No evident changes of cover loss were detected in Punta Salines (Fig. 7). Overall, there was no significant difference regarding taxonomic groups (*X^2^* = 6.9, df = 5, p>0.05) or morphological forms (*X^2^* = 9.8, df = 5, p>0.05). The massive and robust sponges *Chondrosia reniformis* and *Agelas oroides* appeared to be less affected, showing approximately 5% cover loss at Carall Bernat and Tascó Petit and no change at Medallot. After the storm, The removal of sessile organisms on the boring sponge *Cliona sp. during the storm* increased the exposed area by 45% at Carall Bernat and +20% at Medallot, respectively (Fig. 7).

## Discussion

The storm of December 26^th^ 2008 was considered to be the strongest recorded in the last 50 years in the northern part of the Catalan coast (41°N - 42°30′N), with the greatest wave power, the highest wave heights, and the longest duration [41], [39], [45]. Its impact, including abrasion of sediment particles, severely affected the littoral communities in the region, causing mortality of sessile benthic organisms, including some long-lived species (mainly sponges and anthozoans) estimated to be more than 50 years old [53],[32]. Coralligenous outcrops exhibit low dynamics and few changes over time at population and community level in absence of large disturbances [51], [63], [28], [32], which makes the impact of extreme events very important for community dynamics. This is even more important in the Mediterranean Sea, which is considered a hotspot of climate change, where exceptional events such as this storm or heat-waves in summers are predicted to increase [34], [35], [36], [16]. Overall, our results quantified the different effects of this rare, extreme event on the community structure dynamics of long-standing coralligenous outcrops. This study is unique in using high-resolution sampling over time to reveal how extreme events can shift coralligenous outcrops characterized by long-lived species and may be of general interest regarding ecological responses to extreme and unusual climatic events.

### Spatial Patterns of the Storm Impacts

The loss of cover of slow growing benthic species was between 58% and 22% immediately after the storm at two of the studied sites, Carall Bernat and Medallot, respectively (Fig. 5). The damage caused by the storm (with an eastern wind direction) was influenced by aspects of orientation, local habitat profiles, depth, and the presence of boulders. The most impacted site, Carall Bernat (wall facing NE), is located within a narrow channel surrounded by large stone blocks, whereas Medallot (wall facing SW) is located in the most westerly part of an archipelago and is better protected from easterly wave swells. Our data showed that approximately 18 out of 24 species at Carall Bernat were severely affected, showing cover loss values higher than 50% (Fig. 7), accompanied by a significant decrease of perennial species richness (24%) (Fig. 3). Although the impact was local and restricted to the northernmost part of the Catalan coast, the damage was among the highest registered for coralligenous outcrops of the NW Mediterranean Sea. High mortality rates of gorgonian populations have been reported to be up to 10–60% after major episodes of mass mortality, such as those related to positive temperature anomalies in the summers of 1999 and 2003 in the NW Mediterranean Sea [29], [64], [31]. However, these studies did not explore overall community shifts (including changes in calcareous algae, sponges, anthozoans, bryozoans, and tunicates). Other studies quantifying the impacts of severe hurricanes and cyclones on coral reefs have also focused on primary framework corals e.g., [65], [20] and have reported reef losses, with values ranging from 17% to 60% [66], [19], [67], [23], [68], thereby highlighting the importance in terms of the broader community changes.

In this study, the effects of the storm were not found to be uniform and synchronous. Although Carall Bernat was the most impacted site, it showed a low recovery of perennial species after one year (see below), whereas Medallot exhibited little cover loss immediately after the storm (cover loss of approximately 8%) but showed a further decline in the following year (23% cover loss of slow growing species), accompanied by a considerable increase in turf algae (∼ 33%) (Fig. 5). We acknowledge the different responses of benthic community dynamics, which integrate different complex history processes and disturbances, and highlight the complexity of identifying unique, combined and/or synergetic effects of disturbance when most coastal habitats are exposed to multiple stressors [67], [69], [70]. Based on this complexity, our results showed that benthic communities dwelling in rather small areas (less than ∼ 10 km^2^) can exhibit significantly different responses to sudden disturbances.

An abrupt shift in the multivariate structure of coralligenous outcrops after the storm was only observed at the most exposed and impacted site, Carall Bernat, which showed the highest compositional variability in response to the disturbance (Fig. 2). Tascó Petit and Punta Salines did not show any significant change on community structure before and after the storm, indicating no major effects of this severe storm at the sheltered sites. This pattern of greater variability was corroborated by an increase of beta diversity in the perennial species composition after the disturbance (immediately after and in the following year) (Fig. 4). After extreme events such as this storm, post-disturbance variability is expected to be elevated and to persist for a longer period of time relative to pre-disturbance conditions, and this variability will be stabilized more gradually, only after the disturbed state has returned to the baseline condition [71], [72]. Our results demonstrate that this unusual storm produced a mosaic of small remaining survivor patches in the most impacted site and reduced the structural complexity of perennialslow-growing benthic species, creating a seascape habitat exhibiting higher fluctuations in the presence/absence of component species, accompanied by a reduction in the number of species, thus reducing the species pool (a decrease of 8 perennial species) (Figs. 2 and 3). Interestingly, this severe storm appeared to have opposite effects on beta diversity (Fig. 4), such that it increased significantly in Carall Bernat due to a high variability in composition, whereas it declined significantly at Medallot. This increased similarity at Medallot between pre-and post-storm conditions may be explained as a consequence of the change in the relative cover of perennial-slow benthic species (decreasing) and turf algae (increasing). Overall, this change in beta diversity (increase or decrease) was accompanied by a loss of functional groups in Carall Bernat and Medallot (Figs. 6 and 7), with a shift in dominance from encrusting algae and perennial animal species to turf-forming algae. Encrusting calcareous algae are the major contributors to coralligenous outcrops and, together with sponges, cnidarians, bryozoans, and tunicates, are the species that characterize this habitat [28]. Thus, their replacement by turf-forming algae may increase the sensibility to invasion, as some of these algae belong to the most invasive species in the Mediterranean Sea, triggering substantial changes in the structure and dynamics of rocky communities and rendering surfaces inhospitable to the recruitment of native invertebrates [73], [74], [75].

### Species Sensitivity

Species sensitivity showed a gradient regarding the site exposure: from high through intermediate to low values of cover loss at Carall Bernat, Medallot, Tascó Petit, and Punta Salines (Fig. 7). Our findings indicated that the damage across perennial species was uneven and those with fragile forms, irrespective of their morphology, were the most impacted, showing cover losses between 50 and 100% (Fig. 7). These results agree with the general observation that fragile branching and foliose corals are the most susceptible to hurricane damage to coral reefs [19], [67], as well as in the NW Mediterranean Sea, a severe winter storm caused high mortality of the fragile bryozoan *Pentapora facialis* [76]. In the present study, the species ranged from short-lived perennial species with estimated ages of 2–5 years (e.g., the crustose coralline alga *Peyssonnelia sp*., the massive-ropy fragile sponge *Clathrina clathrus*, and the delicate tree-like bryozoans *Adeonella calveti* and *Myriapora truncata* to persistent and long-lived perennial species with estimated longevities of 50–100 years (e.g., the gorgonian *Paramuricea clavata*, the scleractinians *Leptopsammia pruvoti* and *Caryophyllia inornata*, and the alcyonacean *Alcyonium acaule)* and encrusting calcareous algae (e.g., *Lithophyllum stictaeforme and Mesophyllum alternans)* with low natural adult mortality [53], [28], [37], [32]. Thus, this unusual event produced high episodic mortality of adults in a community in which this rarely occurs under natural conditions. These observations are in agreement with the fact that large and infrequent disturbances such as this storm are considered to drive species interactions and community dynamics, which cause long-term effects on both marine and terrestrial communities [6], [77], [5], [67].

### Patterns of Surviving Patches

The strong abrasive effect of the storm did not completely homogenize the available space by creating a seascape of bare substrate at the most impacted site; rather, it produced a mosaic of small remaining surviving patches of perennial benthic species (with values of perennial-slow growing species cover ranging from 10% to 50%), associated with a decrease of habitat complexity and heterogeneity. Spatial heterogeneity following large disturbances has been widely documented in both marine and terrestrial ecosystems [77], [78], [79], and it has been recognized that biotic residuals (e.g., surviving roots and rhizomes of plants, as well as fragments of corals and sponges) are regularly available, even following a large disturbance [80], [81], [82]. In the present study, the existence of small patches after the storm (mainly encrusting algae and clonal animals such as encrusting sponges, anthozoans, and tunicates) at the most impacted site was fundamental for slight recovery, with a minor increase of cover being observed during the following year. This increase of perennial-slow growing species represented an increase of 10% (37% ±3.9) immediately after the impact (2009) to 46% ±4.7 (2010) (Fig. 5). We hypothesize that these surviving colonies and fragments favored faster recovery via vegetative regrowth and this partial recovery occurred more rapidly than could take place through the growth of new recruits via larvae. Our results showed that one year is not enough to re-establish the community to its prior state before the storm (Figs. 2, 3, 4, 5, and 6). Only two encrusting calcified algae (*Lithophyllum stictaeforme and Mesophyllum alternans)* and six clonal species (the sponges *Crambe crambe*, *Dysidea avara, Corticium candelabrum, Phorbas tenacior*, the anthozoans *Parazoanthus axinellae*, and the tunicate *Cystodytes dellechiajei)* contributed to regrowth from the remnant tissue. The finding of small remaining surviving patches is of special interest to understand community responses due to the overall low dynamics of coralligenous species combined with the infrequent or unsuccessful recruitment events recorded for sexually produced larvae of clonal organisms [83], [37], [32].

### Conclusion

With the increasing threat to coastal habitats due to global warming and other interacting factors, there is growing concern about the capacity of ecosystems to absorb multiple disturbances occurring over short time periods [8], [10], [11]. Global warming is predicted to increase the frequency and magnitude of extreme climate and weather events [2], [16]. For the western Mediterranean Sea, a decrease in the total number of cyclones has been predicted [84], but an increase of wind and wave intensity [85], [86]. Consequently, the observed damage makes it evident that recurrent severe storms will seriously affect coralligenous outcrops, posing threats to their resilience. Based on the complex responses to disturbance, efforts to acquire and analyze data over time are fundamental to quantify these changes and evaluate the ecological mechanisms behind them, which will ultimately allow us to develop our capacity to predict long-term and larger scale community shifts. The effects of this storm were difficult to predict, but now that they have been registered, they provide new insights into population and community dynamics. Consequently, under the present warming scenario and due to the high diversity that the Mediterranean Sea harbors [87], [26], we emphasize the need for long-time empirical and modeling studies on sublittoral benthic communities. This information is crucial not only for understanding the mechanisms underlying the dynamics of these communities and the ecological consequences of global climate change but also for determining effective management and conservation approaches to maintain the biodiversity of the Mediterranean.

## Supporting Information

Figure S1 **Non-metric multidimensional scaling (NMDS) based on the Bray-Curtis resemblance measure for species presence/absence data from 2006 to 2010.** A 4- group model was obtained by SIMPROF analysis: Tascó Petit (2006–2010); Punta Salines (2008–2009), Medallot (2006–2010) and the pre-storm years of Carall Bernat (2006–2008); the immediate post-storm years (2009–2010) of Carall Bernat and an independent group of Medallot (2007). Each symbol represents 8 photographs analyzed.
(TIF)

Table S1 **List of the taxa identified in this study.** Boring (BOR), Cup (CUP), Encrusting algae (ENA), Encrusting (ENC), Massive (MAS), Pedunculated (PEN), Tree (TREE).
(DOCX)

Table S2 **Cover area (%) of the representative macrobenthic taxa before and after the physical disturbance generated by the storm.** Boring (BOR), Cup (CUP), Encrusting algae (ENA), Encrusting (ENC), Massive (MAS), and Tree (TREE).
(DOCX)

Table S3 **Results of 2-way PERMANOVA based on Bray-Curtis dissimilarity for macrobenthic taxa.** Pair-wise comparisons using permutations of the *t*-statistic for the factor Site and the interaction term Site*Before/After are also indicated.
(DOCX)

Table S4 **Results of 2-way PERMANOVA based on Euclidian distances for the number of species.** Pair-wise comparisons using permutations of the *t*-statistic for the factor Site and Site*BA (Before/After) effects are also indicated.
(DOCX)

Table S5 **Results of 2-way PERMANOVA analyses based on Euclidian distances for the cover area of the principal groups of sessile organisms and bare substrate.** Pair-wise comparisons using permutations of the *t*-statistic for the factor Site and Site*BA (Before/After) effects are also indicated
(DOCX)

Table S6 **Results of 2-way PERMANOVA analyses based on Euclidian distances for the cover of growth forms of sessile species.** Pair-wise comparisons using permutations of the *t*-statistic for the factor Site and Site*BA (Before/After) effects are also indicated
(DOCX)
