# Supplementary figures and images for: Impacts on Coralligenous Outcrop Biodiversity of a Dramatic Coastal Storm
Source: PLoS One. 2013 Jan 10;8(1):e53742. doi: 10.1371/journal.pone.0053742 (PMC3542355; doi:10.1371/journal.pone.0053742)

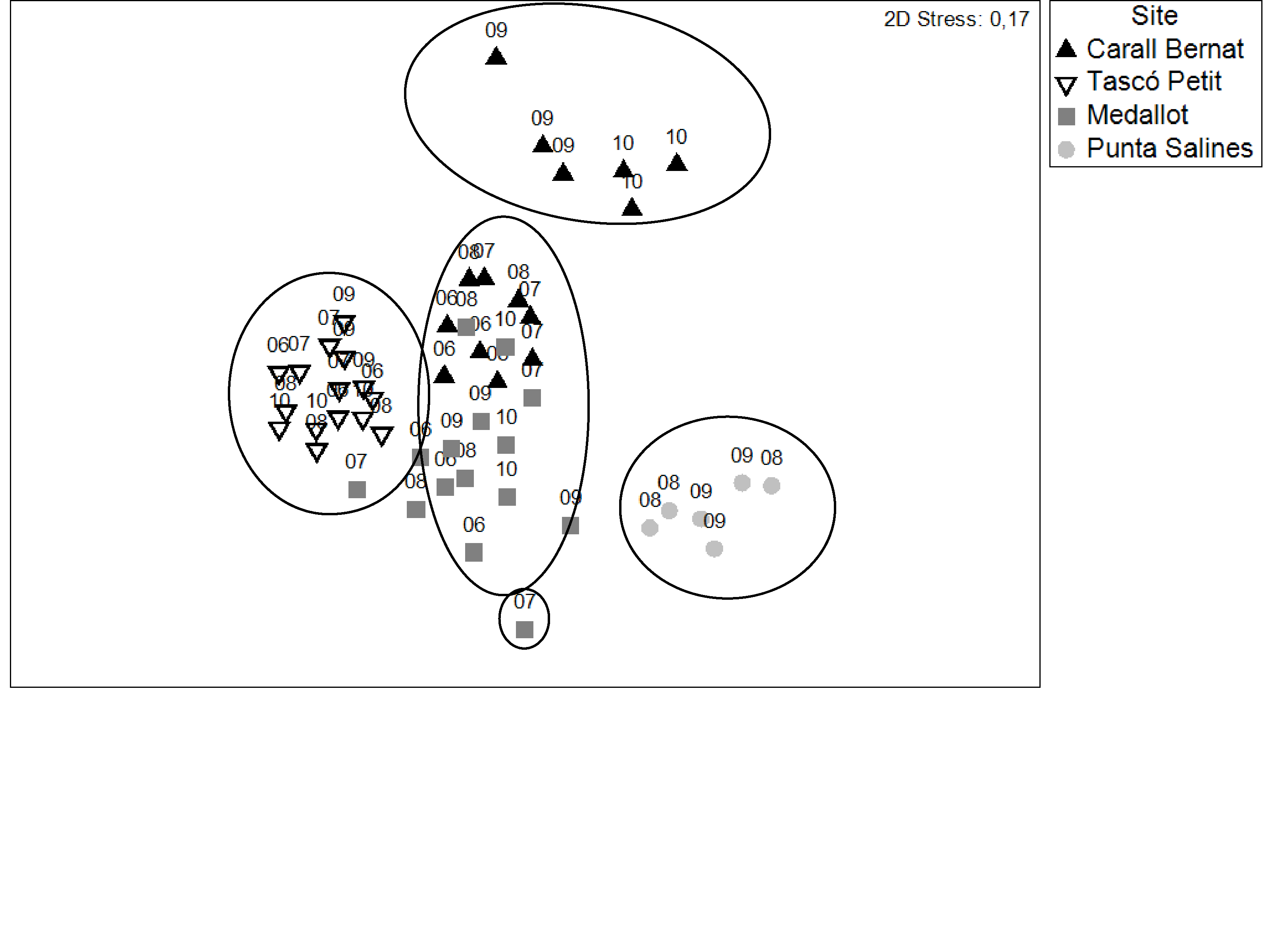

Supplement: Figure S1 — Non-metric multidimensional scaling (NMDS) based on the Bray-Curtis resemblance measure for species presence/absence data from 2006 to 2010. A 4- group model was obtained by SIMPROF analysis: Tascó Petit (2006–2010); Punta Salines (2008–2009), Medallot (2006–2010) and the pre-storm years of Carall Bernat (2006–2008); the immediate post-storm years (2009–2010) of Carall Bernat and an independent group of Medallot (2007). Each symbol represents 8 photographs analyzed. (TIF) [file pone.0053742.s001.tif]
