# Supplementary material for: Impacts on Coralligenous Outcrop Biodiversity of a Dramatic Coastal Storm
Source: PLoS One. 2013 Jan 10;8(1):e53742. doi: 10.1371/journal.pone.0053742 (PMC3542355; doi:10.1371/journal.pone.0053742)
Supplement: Table S1 — List of the taxa identified in this study. Boring (BOR), Cup (CUP), Encrusting algae (ENA), Encrusting (ENC), Massive (MAS), Pedunculated (PEN), Tree (TREE). (DOCX) [file pone.0053742.s002.docx]

**Table S1. List of the taxa identified in this study.** Boring (BOR), Cup (CUP), Encrusting algae (ENA), Encrusting (ENC), Massive (MAS), Pedunculated (PEN), Tree (TREE).

| **Taxa** | Growth Form | Carall Bernat | Tascó Petit | Medallot | Punta Salines |
| --- | --- | --- | --- | --- | --- |
| **Chlorophyta** | | | | | |
| *Flabellia petiolata* | PEN | + | + | + | + |
| *Halimeda tuna* | PEN | - | - | + | - |
| *Palmophyllum crassum* | ENA | + | - | + | - |
| *Valonia macrophysa* | ENC-MAS | + | - | + | - |
| **Rhodophyta** | | | | | |
| *Lithophyllum stictaeforme** | ENA | + | + | + | + |
| *Mesophyllum alternans ** | ENA | + | + | + | + |
| *Peyssonnelia* sp.*** | ENA | + | + | + | + |
| **Porifera** | | | | | |
| *Acanthella acuta** | MAS | + | + | + | + |
| *Agelas oroides** | MAS | + | + | + | + |
| *Axinella damicornis** | MAS | + | + | + | + |
| *Cacospongia* sp. | MAS | + | + | + | - |
| *Chondrosia reniformis** | MAS | + | + | + | + |
| *Clathrina clathrus** | MAS | + | + | + | - |
| *Cliona* sp.*** | BOR | + | + | + | + |
| *Corticium candelabrum** | MAS | + | + | + | + |
| *Crambe crambe** | ENC | + | + | + | + |
| *Crella (Grayella) pulvinar** | ENC | + | + | + | + |
| *Dendroxea lenis* | ENC | + | - | - | - |
| *Dictyonella* sp. | ENC-MAS | + | + | + | + |
| *Dysidea avara** | ENC | + | + | + | + |
| *Fasciospongia cavernosa* | ENC | + | + | + | - |
| *Haliclona (Halichoclona) fulva* | ENC | - | + | + | - |
| *Haliclona (Soestella) mucosa* | ENC | + | + | + | - |
| *Haliclona* sp. | ENC | - | + | + | - |
| *Hemimycale columella** | ENC | + | + | + | + |
| *Hexadella racovitzai** | ENC | + | + | + | + |
| *Ircinia oros** | MAS | + | + | + | - |
| *Ircinia fasciculata* | MAS | - | - | + | - |
| *Ircinia variabilis* | MAS | + | + | + | + |
| *Oscarella* sp.*** | MAS | + | + | + | - |
| *Petrosia ficiformis** | MAS | + | + | + | + |
| *Phorbas tenacior** | ENC | + | + | + | + |
| *Phorbas topsenti* | ENC | - | - | + | - |
| *Pleraplysilla spinifera** | ENC | + | + | + | + |
| *Prosuberites longispinus* | ENC | + | + | + | - |
| *Raspaciona aculeata* | ENC | + | + | - | + |
| *Spirastrella cunctatrix** | ENC | + | + | + | + |
| *Spongia (Spongia) officinalis* | MAS | + | + | - | - |
| *Spongia virgultosa* | MAS | + | - | + | - |
| *Terpios granulosa* | ENC | + | + | - | - |
| **Anthozoa** | | | | | |
| *Alcyonium acaule** | MAS | + | + | + | + |
| *Alcyonium coralloides* | ENC | + | + | + | + |
| *Caryophyllia inornata** | CUP | + | + | + | + |
| *Corallium rubrum* | TREE | - | + | - | - |
| *Hoplangia durotrix* | CUP | + | + | - | - |
| *Leptopsammia pruvoti** | CUP | + | + | + | + |
| *Paramuricea clavata** | TREE | + | + | + | + |
| *Parazoanthus axinellae** | ENC | + | + | + | + |
| **Polychaeta** | | | | | |
| *Filograna implexa / Salmacina dysteri** | TREE | + | + | + | + |
| *Protula* sp./ *Serpula vermicularis* | ENC | + | + | + | - |
| Serpulidae | ENC | + | + | + | - |
| **Bryozoa** | | | | | |
| *Adeonella calveti*/*Smittina cervicornis** | TREE | + | + | + | + |
| *Beania magellanica* | ENC | - | + | + | - |
| *Chartella* *tenella* | TREE | - | + | + | + |
| *Margaretta cereoides* | TREE | - | - | + | - |
| *Myriapora truncata** | TREE | + | + | + | + |
| *Pentapora fascialis* | TREE | - | - | + | - |
| *Reteporella grimaldii** | TREE | - | + | + | + |
| *Schizomavella* sp. | ENC | + | + | + | + |
| *Turbicellepora* sp. | TREE | + | + | + | - |
| **Tunicata** | | | | | |
| *Cystodytes* *dellechiajei** | ENC | + | + | + | + |
| Didemnum sp.1 | ENC | + | - | + | - |
| Didemnum sp. 2 | ENC | + | - | + | - |
| *Halocynthia papillosa** | MASS | + | + | + | + |

* taxa accounted for 90% of the SIMPER analysis
