# Supplementary material for: Impacts on Coralligenous Outcrop Biodiversity of a Dramatic Coastal Storm
Source: PLoS One. 2013 Jan 10;8(1):e53742. doi: 10.1371/journal.pone.0053742 (PMC3542355; doi:10.1371/journal.pone.0053742)
Supplement: Table S2 — Cover area (%) of the representative macrobenthic taxa before and after the physical disturbance generated by the storm. Boring (BOR), Cup (CUP), Encrusting algae (ENA), Encrusting (ENC), Massive (MAS), and Tree (TREE). (DOCX) [file pone.0053742.s003.docx]

**Table S2. Cover area (%) of the representative macrobenthic taxa before and after the physical disturbance generated by the storm.** Boring (BOR), Cup (CUP), Encrusting algae (ENA), Encrusting (ENC), Massive (MAS), Tree (TREE).

nc: no change

| **Taxa** | **Group** | **Carall Bernat** | | **Tascó Petit** | | **Medallot** | | **Punta Salines** | |
| --- | --- | --- | --- | --- | --- | --- | --- | --- | --- |
| **Rhodophyta** |  | Before | After | Before | After | Before | After | Before | After |
| *Lithophyllum stictaeforme* | ENA | 2.3 | 0.3 | 4.5 | 3.3 | 10.0 | 9.0 | 5.5 | nc |
| *Mesophyllum alternans* | ENA | 13.8 | 7.6 | 14.5 | nc | 19.8 | 9.6 | 18.6 | nc |
| *Palmophyllum crassum* | ENA | 1.5 | 0.3 | - | - | 1.3 | nc | - | - |
| *Peyssonnelia* sp. | ENA | 5.5 | 0 | 9.5 | nc | 40.0 | 36.0 | 22.0 | nc |
| **Porifera** |  |  |  |  |  |  |  |  |  |
| *Acanthella acuta* | MAS | 1.16 | 0.16 | 3.5 | 2.3 | 1.3 | nc | 4.5 | nc |
| *Agelas oroides* | MAS | 3.3 | 3.1 | 7.5 | 7 | 6.1 | nc | 1.3 | nc |
| *Axinella damicornis* | MAS | 2 | 0.5 | 2.8 | nc | 3.6 | nc | 3.0 | nc |
| *Chondrosia reniformis* | MAS | 3.2 | 3 | 3 | nc | 3 | nc | - | - |
| *Clathrina clathrus* | MAS | 1.2 | 0 | 5.2 | 1.6 | 0.8 | nc | - | - |
| *Cliona* sp. | BOR | 0.8 | 1.2 | 0.5 | nc | 3.7 | 4.5 | 9.5 | nc |
| *Corticium candelabrum* | MAS | 1.8 | 1.5 | - | - | - | - | - | - |
| *Crella pulvinar* | ENC | - | - | 3.3 | nc | 1 | nc | - | - |
| *Crambe crambe* | ENC | 12.1 | 9.6 | 8 | 8 | 10 | nc | 16.3 | nc |
| *Disydea avara* | ENC | 7.8 | 2.8 | 1.6 | nc | 2 | 1 | 6.0 | nc |
| *Hemimycale columella* | ENC | 0.16 | 0 | 3.2 | nc | 0.5 | nc | - | - |
| *Hexadella racovitzai* |  | - | - | - | - | 4 | 3.3 | - | - |
| *Ircinia oros* | MAS | - | - | 4.8 | nc | 0.8 | nc | - | - |
| *Ircninia variabilis* | MAS | - | - | 3 | 2.8 | 0.3 | nc | - | - |
| *Oscarella sp.* | MAS | - | - | 24.5 | 21.8 | - | - | - | - |
| *Petrosia fisciformis* | MAS | - | - | 1.3 | nc |  |  | - | - |
| *Phorbas tenacior* | ENC | 8.2 | 4.2 | 2.5 | 1.8 | 2 | nc | 5.5 | nc |
| *Pleraplysilla spinifera* | ENC |  |  | 1.5 | nc | 2 | nc | - | - |
| *Spirastrella cunctatrix* | ENC | - | - | 3.3 | nc | 8.6 | nc | - | - |
| **Anthozoa** |  |  |  |  |  |  |  |  |  |
| *Alcyonium acaule* | MAS | 1.6 | 0.9 | 3.6 | nc | 6 | nc | 2.2 | nc |
| *Caryophyllia inornata* | CUP | 0.3 | 0.16 | 2.6 | nc | 1.6 | nc | - | - |
| *Leptopsammia pruvoti* | CUP | 5.5 | 2.6 | 21.5 | nc | 5 | nc | 5 | nc |
| *Paramuricea clavata* | TREE | 8.8 | 2.6 | 12 | 11 | 9.6 | 7.8 | 5 | nc |
| *Parazoanthus axinellae* | ENC | 10.6 | 3.1 | 17.5 | 16.5 | 17.2 | 15.6 | 9 | nc |
| ***Polychaeta*** |  |  |  |  |  |  |  |  |  |
| *Filograna implexa / Salmacina dysteri* | TREE | - | - | 0.3 | 0.1 | 1.8 | 0 | - | - |
| **Bryozoa** |  |  |  |  |  |  |  |  |  |
| *Adeonella calveti*/*Smittina cervicornis* | TREE | 1.7 | 0 | 13 | 10.5 | 6.8 | 5 | 2.5 | nc |
| *Myriapora truncata* | TREE | 1.3 | 0 | 0.6 | nc | 1 | 0 | 1 | nc |
| Reteporella sp. | TREE | - | - | 0.8 | nc | 0.3 | 0.16 | 0.5 | nc |
| **Tunicata** |  |  |  |  |  |  |  |  |  |
| *Cystodytes* | ENC | 4.5 | 2.1 | 10.5 | nc | 9.1 | nc | 10.6 | nc |
| *Halocynthia papillosa* | MAS | - | - | 2 | 0 | 0.3 | nc | 0.3 | nc |
