# Supplementary material for: Impacts on Coralligenous Outcrop Biodiversity of a Dramatic Coastal Storm
Source: PLoS One. 2013 Jan 10;8(1):e53742. doi: 10.1371/journal.pone.0053742 (PMC3542355; doi:10.1371/journal.pone.0053742)
Supplement: Table S3 — Results of 2-way PERMANOVA based on Bray-Curtis dissimilarity for macrobenthic taxa. Pair-wise comparisons using permutations of the t-statistic for the factor Site and the interaction term Site*Before/After are also indicated. (DOCX) [file pone.0053742.s004.docx]

**Table S3.** Results of 2-way PERMANOVA analyses based on Bray-Curtis dissimilarity for macrobenthic taxa. Pair-wise comparisons using permutations of the *t*-statistic for the factor Site and the interaction term Site*Before/After are also indicated

| **Source** | **df** | **SS** | **MS** | **Pseudo_F** | **P** | **Pair-wise comparisons** |
| --- | --- | --- | --- | --- | --- | --- |
| Site | 3 | 9625 | 3208.3 | 20.25 | 0.0001 | Carall Bernat ≠ Tascó Petit  t=5.04 (p<0.0001)  Carall Bernat ≠ Medallot  t=3.66 (p<0.0001)  Carall Bernat ≠ Punta Salines  t=4.964 (p<0.0001)  Tascó Petit ≠ Medallot  t=3.70 (p<0.0001)  Tascó Petit ≠ Punta Salines  t=6.29 (p<0.0001)  Medallot ≠ Punta Salines  t=3.94 (p<0.0001) |
| Before/After | 1 | 543.2 | 543.2 | 1.24 | 0.3894 |  |
| Site*BA | 3 | 1410.5 | 470.17 | 2.96 | 0.0001 | Carall Before ≠ Carall After  t= 2.98 (p<0.0002)  Tascó Petit Before = Tascó Petit After  t= 1.48 (p>0.05)  Medallot Before = Medallot After  t= 1.36 (p>0.05)  Pta Salines Before = Pta Salines After  t= 1.40 (p>0.05) |
| Residual | 43 | 6810.6 | 158.39 |  |  |  |
| Total | 50 | 18539 |  |  |  |  |
|  |  |  |  |  |  |  |
