# Supplementary material for: Impacts on Coralligenous Outcrop Biodiversity of a Dramatic Coastal Storm
Source: PLoS One. 2013 Jan 10;8(1):e53742. doi: 10.1371/journal.pone.0053742 (PMC3542355; doi:10.1371/journal.pone.0053742)
Supplement: Table S4 — Results of 2-way PERMANOVA based on Euclidian distances for the number of species. Pair-wise comparisons using permutations of the t-statistic for the factor Site and Site*BA (Before/After) effects are also indicated. (DOCX) [file pone.0053742.s005.docx]

**Table S4.** Results of 2-way PERMANOVA analyses based on Euclidian distances for the number of species. Pair-wise comparisons using permutations of the *t*-statistic for the factor Site and Site*BA (Before/After) effects are also indicated

| **Permanova** | **df** | **SS** | **MS** | **Pseudo_F** | **P** | **Pair-wise** |
| --- | --- | --- | --- | --- | --- | --- |
| Site | 3 | 998.05 | 332.68 | 34.416 | 0.0001 | Carall Bernat ≠ Tascó Petit t=4.3292, p<0.0001  Carall Bernat ≠ Medallot t=4.7922, p<0.001  Carall Bernat ≠ Pta Salines t=6.5, p<0.001  Tascó Petit = Medallot t=0.108 ; p=>0.05  Tascó Petit ≠ Pta Salines t=8.2, p<0.0001  Medallot ≠ Pta Salines t=9.9, p<0.0001 |
| Before/After | 1 | 34.77 | 34.77 | 0.445 | 0.581 |  |
| Site*BA | 3 | 258.41 | 86.13 | 8.910 | 0.0001 | Carall Before ≠ Carall After  t=5.995; p<0.0009  Tascó Petit Before = Tascó Petit After  t=1.145; p>0.05  Medallot Before = Medallot After  t=0.553; p>0.05  Punta Salines Before = Punta Salines After  t=1.25; p>0.05 |
| Residual | 43 | 415.67 | 9.66 |  |  |  |
|  |  |  |  |  |  |  |
