# Supplementary material for: Impacts on Coralligenous Outcrop Biodiversity of a Dramatic Coastal Storm
Source: PLoS One. 2013 Jan 10;8(1):e53742. doi: 10.1371/journal.pone.0053742 (PMC3542355; doi:10.1371/journal.pone.0053742)
Supplement: Table S5 — Results of 2-way PERMANOVA analyses based on Euclidian distances for the cover area of the principal groups of sessile organisms and bare substrate. Pair-wise comparisons using permutations of the t-statistic for the factor Site and Site*BA (Before/After) effects are also indicated (DOCX) [file pone.0053742.s006.docx]

**Table S5.** Results of 2-way PERMANOVA analyses based on Euclidian distances for the cover area of the principal groups of sessile organisms and bare substrate. Pair-wise comparisons using permutations of the *t*-statistic for the factor Site and Site*BA (Before/After) effects are also indicated

| **Permanova** | **df** | **SS** | **MS** | **Pseudo_F** | **P** | **Pair-wise** |
| --- | --- | --- | --- | --- | --- | --- |
| Site | 2 | 8030.4 | 4015.2 | 12.08 | 0.0001 | Carall Bernat ≠ Tascó Petit t=5.3038, p<0.0001  Carall Bernat ≠ Medallot t=2.39, p<0.005  Tascó Petit ≠ Medallot t=5.30 ; p<0.0001 |
| Before/After | 1 | 8017.6 | 8017.6 | 2.25 | 0.266 |  |
| Site*BA | 2 | 7102.4 | 3551.2 | 10.69 | 0.0001 | Carall Before ≠ Carall After  t=4.085; p<0.001  Tascó Petit Before = Tascó Petit After  t=2.63; p>0.05  Medallot Before ≠ Medallot After  t=3.107; p<0.001 |
| Residual | 39 | 12953 | 333.14 |  |  |  |
|  |  |  |  |  |  |  |
