# Supplementary material for: Impacts on Coralligenous Outcrop Biodiversity of a Dramatic Coastal Storm
Source: PLoS One. 2013 Jan 10;8(1):e53742. doi: 10.1371/journal.pone.0053742 (PMC3542355; doi:10.1371/journal.pone.0053742)
Supplement: Table S6 — Results of 2-way PERMANOVA analyses based on Euclidian distances for the cover of growth forms of sessile species. Pair-wise comparisons using permutations of the t-statistic for the factor Site and Site*BA (Before/After) effects are also indicated (DOCX) [file pone.0053742.s007.docx]

**Table S6.** Results of 2-way PERMANOVA analyses based on Euclidian distances for the cover of growth forms of sessile species. Pair-wise comparisons using permutations of the *t*-statistic for the factor Site and Site*BA (Before/After) effects are also indicated

| Permanova | df | SS | MS | Pseudo_F | P | Pair-wise |
| --- | --- | --- | --- | --- | --- | --- |
| Site | 2 | 10006 | 5003 | 113.26 | 0.0001 | Carall Bernat ≠ Tascó Petit t=12.7, p<0.0001  Carall Bernat ≠ Medallot t=9.1, p<0.001  Tascó Petit ≠ Medallot t=10.60 ; p<0.0001 |
| Before/After | 1 | 945.1 | 945.1 | 5.76 | 0.104 |  |
| Site*BA | 2 | 327.8 | 163.9 | 3.71 | 0.0063 | Carall Before ≠ Carall After  t=4.76; p<0.01  Tascó Petit Before = Tascó Petit After  t=2.13; p>0.05  Medallot Before = Medallot After  t=2.45; p>0.05 |
| Residual | 9 | 397.5 | 44.1 |  |  |  |
|  |  |  |  |  |  |  |
